# Post-transplantation cyclophosphamide combined with tacrolimus and low-dose post-engraftment anti-thymoglobulin as GVHD prophylaxis for patients undergoing peripheral blood stem cell transplantation from haploidentical family donor: A single center analysis

**DOI:** 10.3389/fmed.2023.1140217

**Published:** 2023-03-31

**Authors:** Wen-hui Gao, Jia-yan Zhu, Li-ning Wang, Ming Wan, Ling Wang, Raynier Devillier, Jie-ling Jiang, Didier Blaise, Jiong Hu

**Affiliations:** ^1^Shanghai Institute of Hematology, Blood and Marrow Transplantation Center, Department of Hematology, Collaborative Innovation Center of Hematology, Rui Jin Hospital, Shanghai Jiao Tong University School of Medicine, Shanghai, China; ^2^Shanghai Clinical Research Center (SCRC), Fenglin International Centre, Shanghai, China; ^3^Department of Hematology, Program of Transplantation and Cell Therapy, Program of Leukemia, Centre de Recherche en Cancérologie de Marseille (CRCM), Institut Paoli-Calmettes, Aix Marseille University, Marseille, France

**Keywords:** PTCy, GVHD, haplo donors, peripheral blood stem cell transplantation, anti-thymocyte globulin

## Abstract

**Introduction:**

Post-transplantation cyclophosphamide (PT-Cy) use is a recent graft-versus-host disease (GVHD) prophylaxis strategy for patients undergoing allogeneic stem cell transplantation (allo-HSCT). PT-Cy combined with two immunosuppressants is now widely used after haplo-identical (haplo) and HLA-matched peripheral blood stem cell (PBSC) transplantations with promising GVHD and relapsefree survival (GRFS) probabilities. Although appealing, these results may benefit from improvement notably outside matched sibling donor transplantation, and should be investigated in various ethnic populations.

**Methods:**

Therefore, we report our experience of GVHD prophylaxis regimen combining PT-Cy and tacrolimus with addition of post-engraftment low-dose anti-thymocyte globulin (ATG) in allogeneic stem cell transplantation from haplo-identical donors (Haplo). Sixtyseven patients were included in the analysis. All patients received myeloablative or intensified sequential conditioning regimen.

**Results:**

The median follow-up was 521 (range, 10~991) days. The cumulative incidences of 100-day grade II-IV acute GVHD was 14.9±4.4%, and no case of grade III-IV acute GVHD was documented. The cumulative incidences of 2-yearchronic GVHD and moderate-to-severe chronic GVHD were 25.4±5.4% and 11.9±4%, respectively. The non-relapse mortality at day+100 and 2year were 7.5±3.2% and 9.0±3.5%, respectively. The cumulative incidence of relapse at 2year was 16±6.4%. The 2-year probability of DFS and OS were 73.8% (95%CI, 61.5~88.4%) and 72.5% (95% CI, 57.1~92.1%), respectively. The 2-year GRFS was estimated as 63.6% (95%CI, 50.6~80%).

**Discussion:**

Our results suggested that a combination of PT-Cy, tacrolimus, and low-dose post-engraftment ATG was a promising GVHD prophylaxis with low incidence of acute GVHD in the haplo-transplantation setting.

## Introduction

Graft-versus-host disease (GVHD) remains as a major complication of allogeneic hematopoietic stem cell transplantation (allo-HSCT) (1). The standard GVHD prophylaxis strategy is mostly based on the use of calcineurin inhibitors (CNI) or combined with other immunosuppressants (IS) (2, 3). Recently, the post-transplantation cyclophosphamide (PT-Cy) regimen has become an important approach for GVHD prophylaxis, not only in the context of allo-HSCT from HLA-matched related donors (MSD) or HLA-matched unrelated donors (MUD) but also from haplo-identical donors (Haplo) (3–6).

Although PT-Cy can be used as a single agent for GVHD prophylaxis in allogeneic bone marrow transplantation (BMT) from MSD donor (7–9), the addition of other IS seems essential in other situations, especially when using peripheral blood stem cells (PBSCs) (10). Ruggeri et al. (10) documented that there was no difference in the risk of grade II-IV acute GVHD (aGVHD) between PT-Cy only and PT-Cy combined with other IS. However, the incidence of extensive chronic GVHD (cGVHD) was significantly higher among patients receiving PT-Cy only (18%) or PT-Cy with one other IS (20%) than those receiving PT-Cy with two other IS (8.5%). Furthermore, PT-Cy with two additional IS did not affect the incidence of relapse or NRM but improved both OS and GRFS. Besides, addition of anti-thymoglobulin (ATG) prior to transplantation did not affect the relapse rate in the multivariate analysis (10).

In a previous phase II clinical trial, we reported that the incidences of grade II-IV aGVHD and moderate-to-severe cGVHD (mod/sev cGVHD) were 39 and 24%, respectively, in adult patients with lymphoid malignancies receiving PT-Cy and CNI as GVHD prophylaxis, using mobilized PBSCs as grafts (11). To further reduce the incidence of aGVHD, we added a single dose of 2.5 mg/kg ATG after documentation of neutrophil engraftment in case of MUD or Haplo donors. Initial analysis showed a low incidence (9.1%) of grade II-IV aGVHD as previously reported (12).

In this study, we analyzed the outcomes of patients included in three prospective clinical trials (NCT 04118075, NCT 04269811, and NCT 04897139) using the same GVHD prophylaxis regimen focusing on the aGVHD in patients with haplo donor transplantation.

## Patients and methods

### Patient selection and eligibility criteria for allo-HSCT

This study was approved by the Human Ethics Committee of Rui Jin Hospital. It was conducted in accordance with the Declaration of Helsinki. The patients were treated at the Blood and Marrow Transplantation Center, Rui Jin Hospital, Shanghai Jiao Tong University School of Medicine. The first 67 adult patients consecutively enrolled in these clinical trials were included in the analysis. The eligibility criteria for allo-HSCT were as follows: (1) adult patients (18–65 years) with hematological malignancies, including acute leukemia, chronic myelomonocytic leukemia(CMML), Myeloid sarcoma, lymphoma, and high-risk MDS; (2) patients receiving their first allo-HSCT from Haplo donors; (3) patients with ECOG ≤3, normal renal and hepatic function (serum creatinine ≤133 μmol/L, serum bilirubin ≤34 μmol/L, serum alanine aminotransferase or aspartate aminotransferase <3 times the upper normal limit), cardiac left ventricular ejection fraction ≥50%, and normal pulmonary function tests (including forced expiratory volume in 1 min) at transplantation; (4) negative serology for hepatitis B, C, and human immunodeficiency virus; and (5) patients providing written informed consent prior to allo-HSCT.

### Conditioning and GVHD prophylaxis regimen

**Figure 1:**
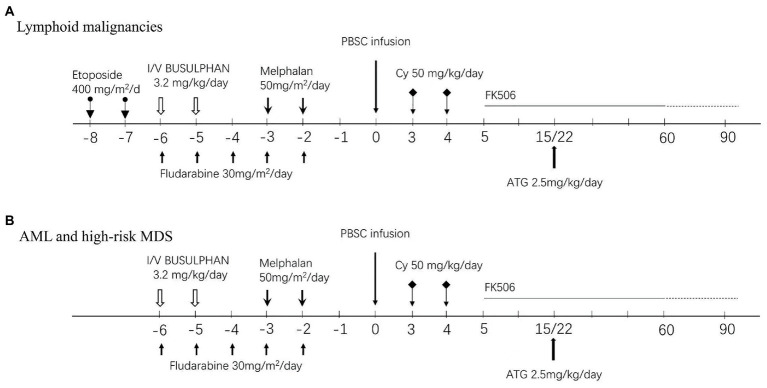
Conditioning regimens and GVHD prophylaxis protocol. **(A)** Lymphoid malignancies. **(B)** AML/high-risk MDS and CMML/Myeloid sacroma.

For lymphoid malignancies, patients received etoposide 400 mg/m^2^/d from day −8 to day −7, fludarabine (FLU) 30 mg/m^2^/d from day −6 to day −2, intravenous busulfan (BU) at 1.6 mg/kg every 12 h in six doses over 3 days (from day −6 to day −5) and melphalan (Mel) 50 mg/m^2^/d from day −3 to day −2. For AML and high-risk MDS, patients received FLU 30 mg/m^2^/d from day −6 to day −2, melphalan (Mel) 50 ~ 70 mg/m^2^/d from day −3 to day −2, and intravenous BU at 1.6 mg/kg every 12 h in four doses over 2 days (from day −6 to day −5). For relapse or refractory AML, patients initially received CLAE chemotherapy as debulking treatment, which consisted of 5 mg/m^2^/d cladribine and 1.5 g/m^2^/day cytarabine for 5 days (day −20 to day −16) and etoposide 200 mg/m^2^/d for 3 days (from day −16 to day −14). With an interval of 7 days, patients received a reduced conditioning regimen of FLU 30 mg/m^2^/day from day −6 to day −2 and intravenous BU at 1.6 mg/kg every 12 h in six doses over 3 days (from day −6 to day −4). In patients with ECOG ≥2, the doses of BU and Mel were reduced accordingly, as shown in Figure 1. BU was administered by infusion over 3 h, and the dose of BU was based on actual body weight (ABW) or adjusted ideal body weight (AIBW, AIBW = ideal body weight (IBW) + 25% (ABW-IBW)) for overweight patients. Granulocyte colony-stimulating factor (G-CSF)-mobilized PBSCs were infused on day 0. GVHD prophylaxis consisted of cyclophosphamide 50 mg/kg on day +3 and day +4, followed by tacrolimus 1.5 mg twice daily, starting on day 5 adjusted with target therapeutic trough level of 5 ~ 15 ng/ml. A single dose of 2.5 mg/kg ATG was administered in patients on day +15 after documentation of neutrophil engraftment. If neutrophil engraft was delayed, the dose of ATG was postponed to day +22 or afterwards likewise, as shown in Figure 1.

### Supportive care

In the case of grade II-IV aGVHD, first line treatment was methylprednisolone 1 ~ 2 mg/kg/day (i.v.) only. If no response was observed, ruxolitinib or basiliximab was added as second line treatment. For cytomegalovirus (CMV) prophylaxis, ganciclovir was administered on day −9 to day −2. After transplantation, pre-emptive treatment of ganciclovir was based on weekly monitoring and administered if CMV DNAemia level > 1 × 10^4^ IU/mL. Additionally, Epstein–Barr virus (EBV) DNA was monitored weekly, and preemptive rituximab was used if EBV-DNA level was >1 × 10^5^ IU/ml to prevent EBV-associated post-transplantation lymphoproliferative disease (PTLD). Regarding the prophylaxis of invasive fungal disease (IFD), risk adapted escalation strategy was used as described in our previous study (12). All patients without previous probable or proven IFD received fluconazole (i.v.) as IFD prophylaxis in the laminar airflow unit. After patients were discharged from the BMT units, fluconazole (p.o) was administered on day 100 in patients undergoing transplantation from MSD without aGVHD. For all patients with MUD or haplo donors and all patients with aGVHD requiring steroid treatment, the prophylaxis was escalated to voriconazole or posaconazole until day 100. For patients with previous documented probable or proven IFD, caspofungin or voriconazole was administered as secondary prevention at the BMT unit, and voriconazole or posaconazole was used sequentially until day 100. Moreover, sulfamethoxazole was administered every other day, from day of engraftment to 100 days after transplantation to prevent pneumocystis. All other supportive care were according to the standard protocol of Rui Jin Hospital.

### Study endpoints

The primary endpoint of the study was the incidence of grade II-IV aGVHD at day +100. Secondary endpoints were engraftment rate, cumulative incidence of grade III-IV aGVHD and NRM at day +100, cumulative incidence of cGVHD, moderate-to-severe cGVHD, relapse, disease-free survival (DFS), overall survival (OS), and GVHD and relapse-free survival (GRFS) at 2 year. Definitions of neutrophil and platelet engraftment, NRM, relapse, DFS, and OS were previously reported (12). Full donor chimerism was defined as ≥95% leukocytes of donor origin in peripheral blood or marrow samples. Mixed chimerism was defined as >5% but <95% leukocytes of donor origin (13). GRFS events were defined as primary engraftment failure, grade III-IV aGVHD, moderate-to-severe cGVHD, and mild cGVHD requiring systemic immunosuppressive treatment, disease relapse, or death from any cause during follow-up after allo-HSCT.

### Measurement of immune reconstitution

The immune reconstitution in terms of peripheral lymphocytes population test was performed regularly after transplantation. The distributions of peripheral CD3+, CD4+, CD8+, NK and CD19+ cells were analyzed by FACSCanto II flow cytometer (Becton Dickinson, United States) labeled with monoclonal antibodies (FITC/PE/Percp/APC/PE-Cy7/APC-Cy7) and the absolute number of cells were calculated based on peripheral blood count and the percentage of cell populations.

### Statistical analysis

The sample size was calculated using the one arm binomial. The null hypothesis was set as the incidence of grade II-IV GVHD ≥30%, whereas the alternate hypothesis was incidence of grade II-IV GVHD ≤15% with one-sided significance of 0.05 and power of 0.8. The incidences of aGVHD, cGVHD, relapse, and NRM were estimated with cumulative incidence functions. The OS, DFS, and GRFS were calculated using the Kaplan–Meier method. The cumulative incidences of relapse and death of any causes were calculated as competing risks (14). Data on patients who were alive in CR were censored at last follow-up on December 31, 2021. The statistical analyses were performed using SPSS 26.0 and R statistical software.

## Results

### Patients and characteristics

**Figure 2:**
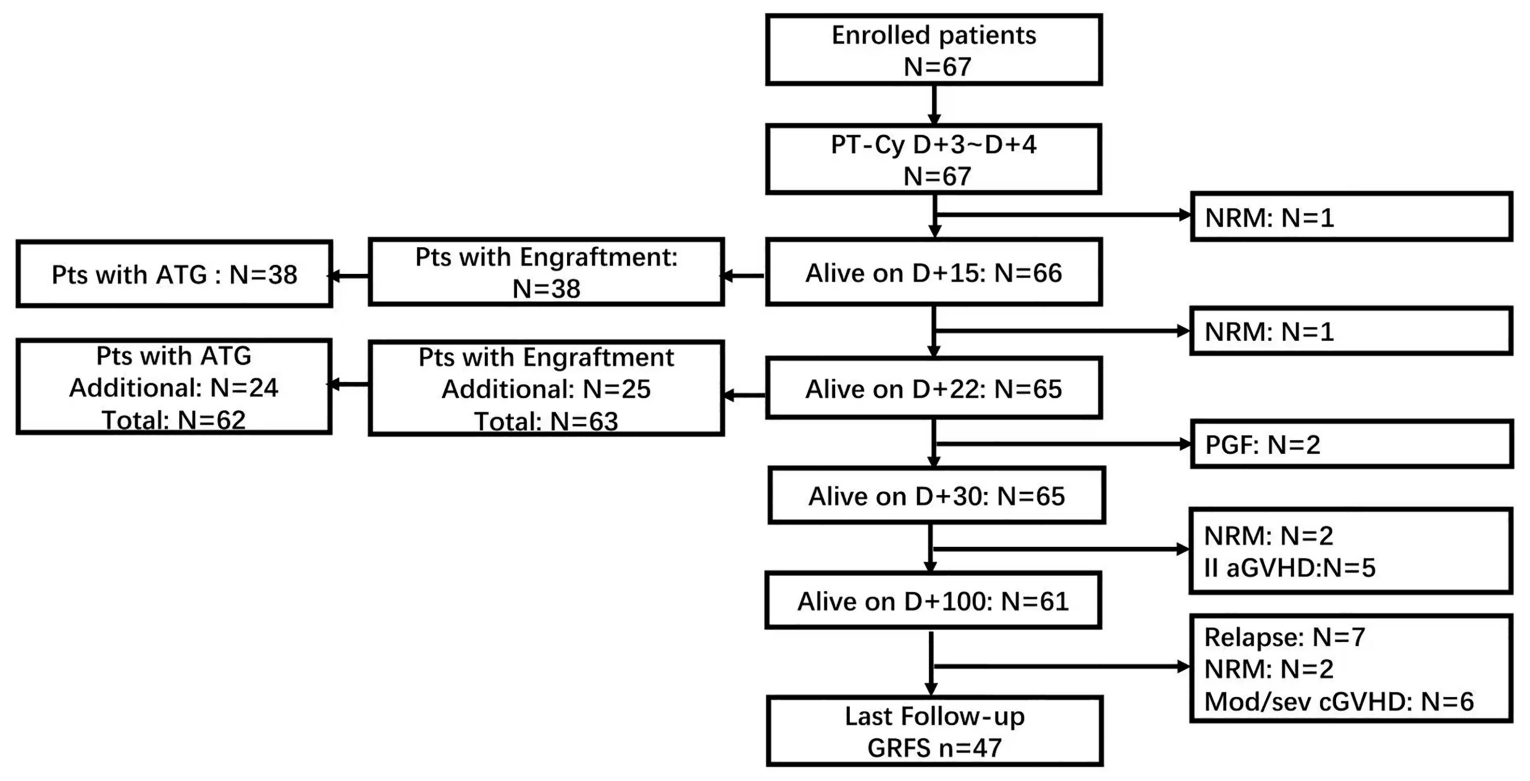
Treatment flowchart.

The demographic data of patients included in the analysis are summarized in Table 1. The median age was 45 years (range, 18 ~ 63 years). Fifty-nine patients had myeloid malignancies, including AML (*n* = 39), high-risk MDS (*n* = 18), chronic myelomonocytic leukemia (CMML, *n* = 1), and myeloid sarcoma (MS, *n* = 1). Eight patients were diagnosed with Lymphoid malignancies acute lymphoblastic leukemia, Hodgkin’s lymphoma at the time of allo-HSCT, 49 patients were in complete remission (*n* = 48) or partial remission (*n* = 1), and 18 patients had active disease. All patients have been followed up for a median of 521 days (range, 10 ~ 991 days); whereas for alive patients, the median follow-up was 527 days (range 319 ~ 991 days). The treatment flow is illustrated in Figure 2.

**Table 1:** Patient and donor characteristics.

	N (% or range)
No. of patients	67
Median follow-up (days, range)	
All patients	521 (10–991)
Alive patients	527 (319–991)
Age (year, median, range)	45 (17–63)
Sex	
Male	41 (61.2%)
Female	26 (38.8%)
Diagnosis	
AML	39 (62.3%)
MDS	18 (24.5%)
CMML	1 (1.9%)
Myeloid sarcoma	1 (1.9%)
ALL	8 (9.4%)
Disease status	
CR	48 (71.6%)
PR	1 (1.5%)
Active	18 (26.9%)
ECOG	
0 – −1	53 (79.1%)
2 – −3	12 (20.9%)
HCT-CI	
0	49 (73.1%)
1 – −2	12 (17.9%)
3 – −4	5 (7.5%)
≥5	1 (1.5%)
DRI	
Low	9 (9.4%)
Intermediate	32 (47.8%)
High/very high	29 (43.3%)
HLA match in Haplo donor	
5/10	39 (58.2%)
6/10	13 (19.4%)
7/10	11 (16.4%)
8/10	3 (4.45%)
9/10	1 (1.5%)
Donor sex	
Male	52 (77.6%)
Female	15 (22.4%)
Median donor age in years (range)	31 (9–62)
Donor or recipient	
Sex matching	
F/M	9 (13.4%)
F/F	6 (9.0%)
M/M	30 (44.8%)
M/F	22 (32.8%)
NK alloreactive donor	26 (56.4%) (28unknown)
Median cells infused (range)	
MNC (´10^8^/kg)	7.29 (4.06 ~ 12.17)
CD34 (´10^6^/kg)	6.31 (3.87 ~ 11.24)
CD3+ (´10^8^/kg)	5.15 (2.68 ~ 7.24)

### Engraftment and chimerism

The median number of mononuclear cells (MNC) and CD34^+^ cells infused were 7.29 × 10^8^/kg (range, 4.06 ~ 13.3 × 10^8^/kg) and 7.87 × 10^6^/kg (range, 3.87 ~ 15.9 × 10^6^/kg), respectively. Two patients died before engraftment (as shown in Figure 2). The engraftment was documented in 63 patients with a median of 15 (range, 11–22) days for neutrophil engraftment and 16 (range, 9–38) days for platelet engraftment, respectively. Among these patients, the chimerism analysis of MNC and CD3^+^ T cells in the bone marrow was performed on day 28 based on the PCR of short tandem repeat (STR) unique for donor or recipient. All patients achieved full donor chimerism (≥99%) both in MNC and CD3^+^ T cells. Two other patients experienced primary graft failure. One patient successfully underwent salvage second allo-HSCT from another haplo-donor, whereas the other patient died of an infection during the work-up for the second allo-HSCT from an alternative donor (Table 2).

**Table 2:** Causes of death.

Causes of death	No.	Description
Relapse	4	rRelapse/refractory AML (day+647); primary refractory T lymphoblastic leukemia in remission with MRD+ after salvage chemotherapy (day +469); Untreated MDS with TP53 mutation(day+723); FLT3-ITD + AML in remission with MRD+(day+622)
Bacteremia	1	MDS not responding to HMA treatment: CRKP colonization, early neutropenic fever with documented of CRKP BSI (day +10)
	1	AML in CR1: CRAB BSI (day +20)
	1	AML in CR1: primary graft failure with prolong neutropenia and documented Stenotrophomonas maltophilia BSI (day +40)
Pneumonia	2	CMML-AML in CR1: pulmonary infection after Cerebral infarction (day +184); Untreated MDS:pulmonary infection (day +337)
Encephalitis	1	ALL in MRD-CR2 after CD19-CAR-T; Pathogen not identified (d + 99)
Heart failure	1	AML in CR1: poor graft function with CMV hemorrhagic cystitis and intractable pericardial effusion (day +88).

### Immune reconstitution

**Figure 3:**
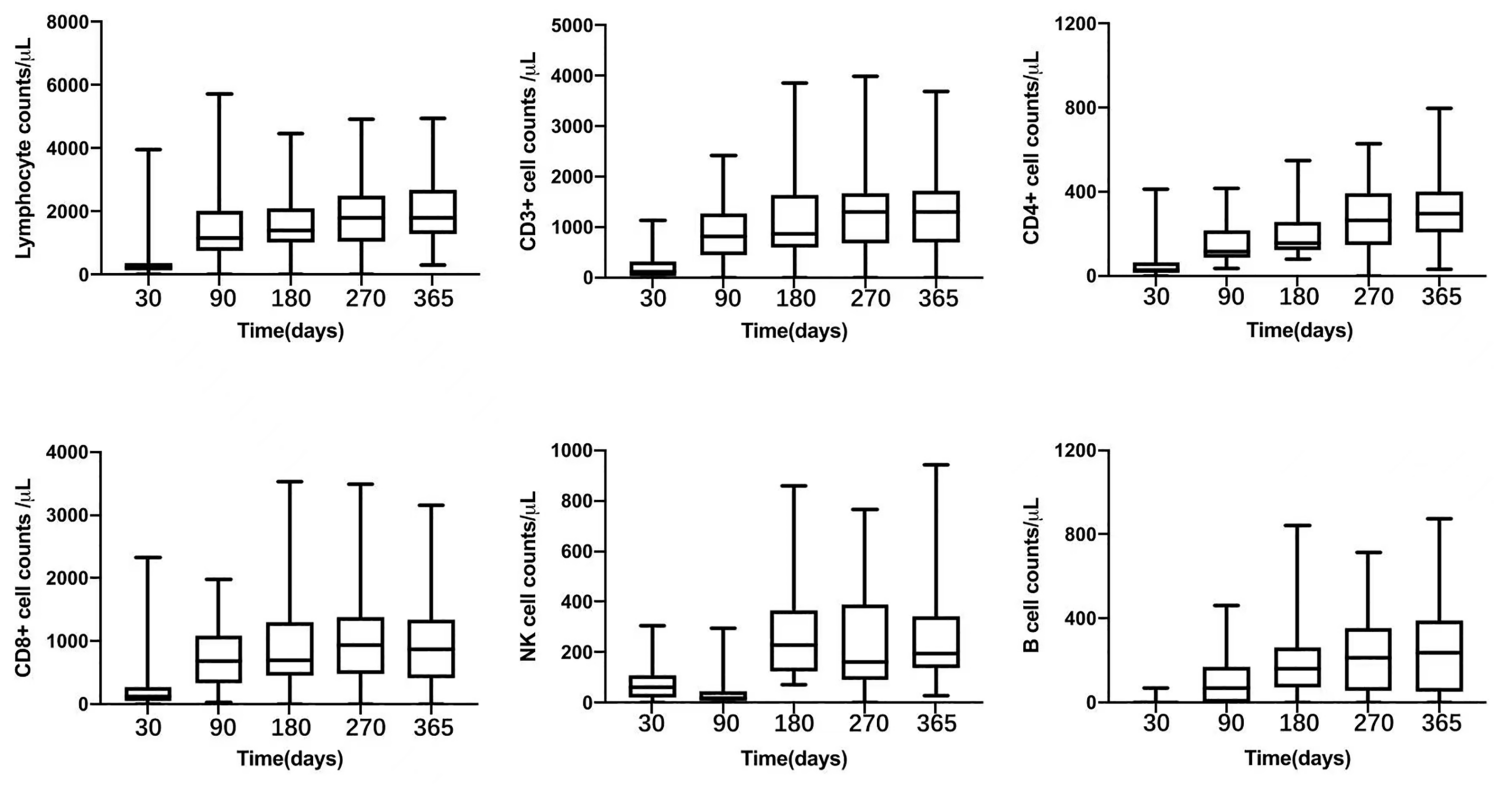
Peripheral lymphocyte counts after transplantation: total lymphocyte oucnt, CD3+, CD4+, CD8+, NK cell, and B lymphocyte.

Peripheral blood lymphocytes were detected at fixed time-points. On day +180 and + 270, median CD3+, CD4+, CD8+, CD56/CD16+ and CD19+ cell counts were 918 (352–3,856) and 1,308(201–3,996), 156 (79–549) and 265 (48–628), 703 (221–3,525) and 976 (150–3,495), 277 (71–860) and 193 (22–1,196), 186 (12–843) and 235 (2–715)/μl, respectively. Since +270 days after transplantation, the median of CD4 + cell counts were above 200/μL and reached nearly 400/μL on +365 days (as shown in Figure 3).

### Acute GVHD

For GVHD prophylaxis, five patients did not receive ATG due to early death before engraftment (*n* = 2), primary engraftment failure (*n* = 2), and multiple complications at engraftment, including persistent fever with unknown origin and progressive hemorrhagic cystitis (*n* = 1). Based on the time of neutrophil engraftment, 62 patients received ATG, as planned, on day+15 (*n* = 38) or day+22 (*n* = 24).

**Figure 4:**
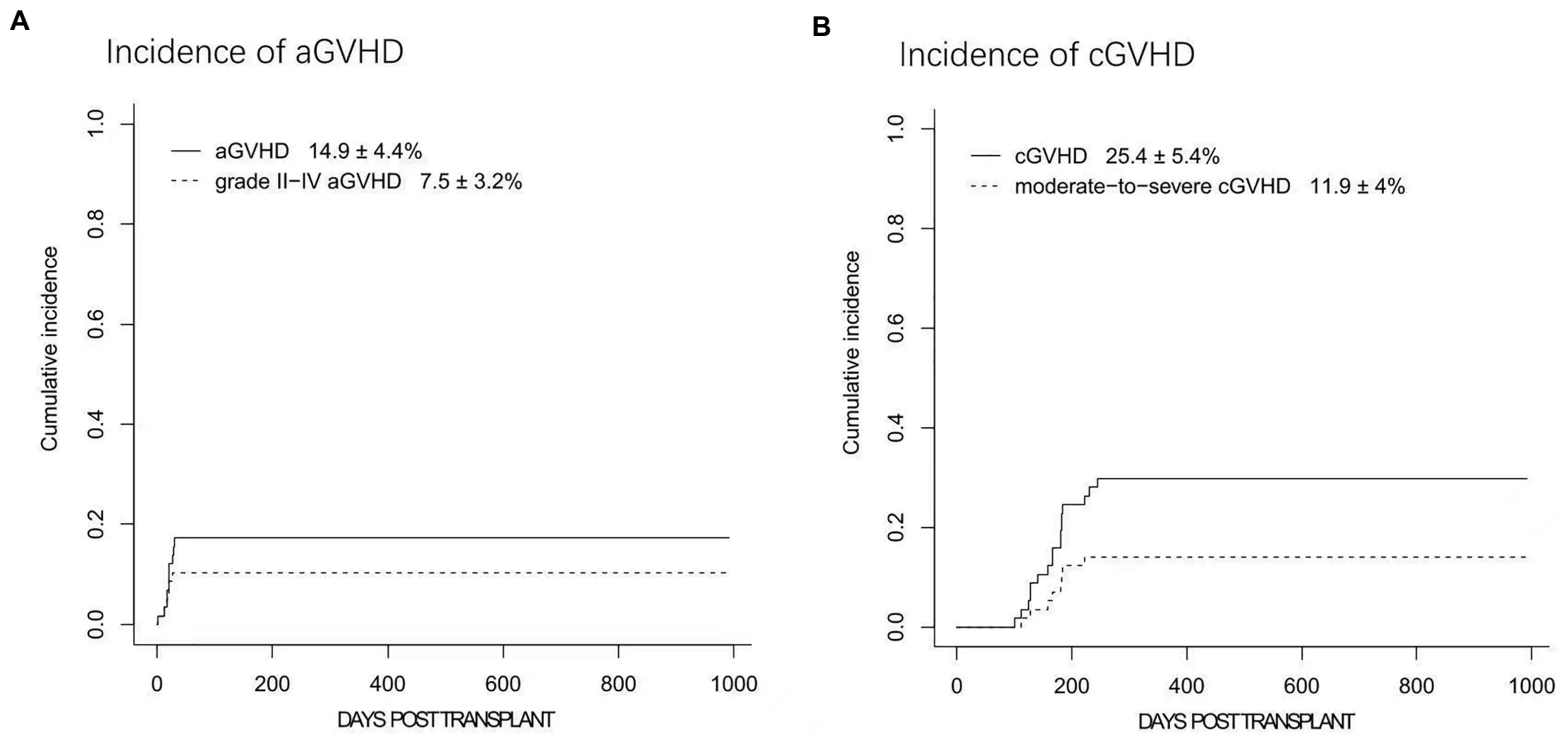
The cumulative incidences (CIs) of acute graft-versus-host disease (aGVHD) and chronic graft-versus-host disease (cGVHD). **(A)** The CIs of grades I-IV aGVHD and grades II-IV aGVHD. **(B)** The 2-year CIs of cGVHD and moderate to severe cGVHD.

Overall, four and four patients developed grade I and II aGVHD, respectively. Therefore, the cumulative incidences of all grade and II-IV aGVHD at day+100 were 14.9 ± 4.4% and 7.5 ± 3.2%, respectively (Figure 4A). No cases of grade III-IV aGVHD were documented. Among four patients with grade II aGVHD, one patient had isolated skin aGVHD, two patients had isolated gut involvement, and another one patient had both skin and gut involved. Notably, all these patients achieved complete response after standard dose corticosteroid treatment (methylprednisolone with 1 ~ 2 mg/kg/d), and no steroid-resistant GVHD was documented.

### Chronic GVHD

Nine patients developed mild cGVHD with skin or mucosa involvement. Mod/sev cGVHD was documented in 8 patients. Among them, 7 patients had skin, liver and mucosa involved, whereas the other patient was diagnosed with bronchiolitis obliterans (BO) together with skin and liver cGVHD. Seven patients with moderate cGVHD responded to systemic corticosteroids combined with tacrolimus or sirolimus therapy. The patient with BO remained well with steroid and tacrolimus therapy after pulmonary transplantation. Overall, the 1-year incidences of cGVHD and mod/sev cGVHD were 25.4 ± 5.4% and 11.9 ± 4.0%, respectively (Figure 4B) while the 2-year cGVHD remained as 25.4 ± 5.4%.

### Infectious complications after allo-HSCT

Most patients experienced febrile neutropenia and were well controlled after hematological reconstitution. Eleven patients had documented pneumonia: five patients with possible aspergillus pneumonia, three patients with bacterial pulmonary infection, and one patient with pneumocystis carinii pneumonia. Within 100 days, CMV reactivation was observed in 34 (50.7%) patients, and five cases of CMV disease were documented, which included two cases of pneumonia and three cases of hemorrhagic cystitis. The median time to CMV reactivation was 40 (range, 12 ∼ 90) days post-transplantation. EBV reactivation was documented in 4 (6.0%) patients on days+40, +41, +102 and + 287, respectively, after transplantation. All four patients responded to dose reduction of tacrolimus (*n* = 2) or recovered spontaneously without any specific intervention (*n* = 2). No EBV-associated PTLD was documented. Besides, four (6.0%) patients developed hemorrhagic cystitis; three of these cases were due to CMV and the other was due to BK virus (Table 3).

**Table 3:** Infectious complications after HSCT.

	*N (%)*
CMV viremia	34 (50.7)
CMV disease	5 (6)
EBV viremia	4 (6.0)
Hemorrhagic cystitis	4 (6)
Pneumonia	
Fungal	5 (7.5)
Bacteria	3 (4.5)
CMV	2 (3.0)
Pneumocystis carinii pneumonia	1 (1.5)

### Overall outcomes

**Figure 5:**
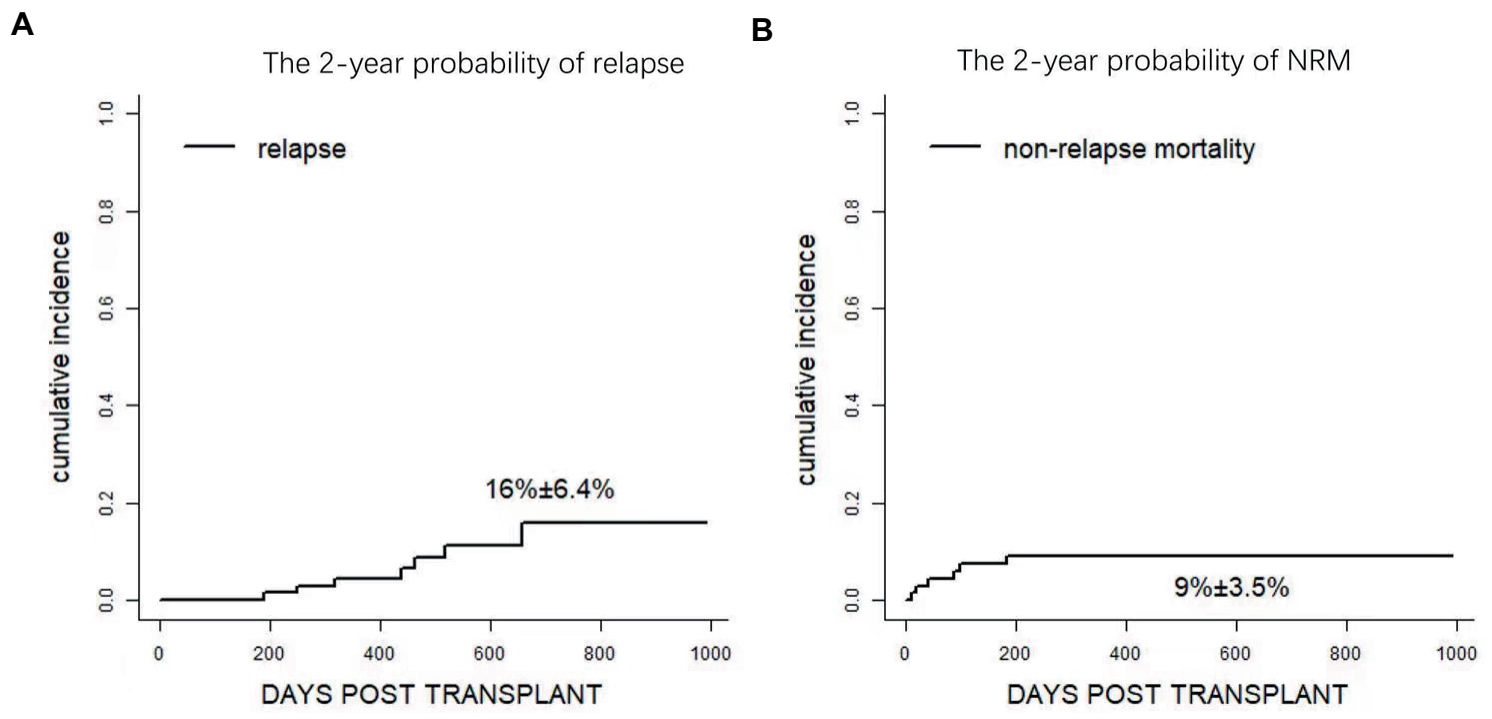
Clinical outcomes after Haplo-PBSCT. **(A)** The 2-year probability of relapse. **(B)** The 1 year probability of non-relapse mortality (NRM).

At the last follow-up, seven patients relapsed. The cases included refractory T lymphoblastic leukemia/lymphoma transplanted in remission after salvage chemotherapy (*n* = 1, day+190), CR1 FLT3-ITD + AML (*n* = 1, day+249), refractory/relapse AML (*n* = 2, day+463 and + 657), and MDS-EB1 with TP53 mutation (*n* = 1, day+516), high risk AML (*n* = 1,day+318), high risk ALL (*n* = 1, day+437). Therefore, the 1-year cumulative incidence of relapse (CIR) was 4.5 ± 2.5% while the overall 2-year CIR was 16% ± 6.4% (Figure 5A and Supplementary Table S1). Eventually, four patients died of disease, and the other three patients were undergoing treatment with Inotuzumab-ozogamicin and second allo-HSCT as salvage therapy.

Regarding NRM, a total of 7 patients died of transplantation-related complications, including bacterial infection in neutropenia (*n* = 3), viral encephalitis (*n* = 1), heart failure (*n* = 1), and pulmonary infection (*n* = 2, as shown in Table 2). NRM at day+100 and 2 year were 7.5 ± 3.2% and 9.0 ± 3.5%, respectively (Figure 5B and Supplementary Table S1). Notably, no patient died of aGVHD or related complications at the last follow-up.

**Figure 6:**
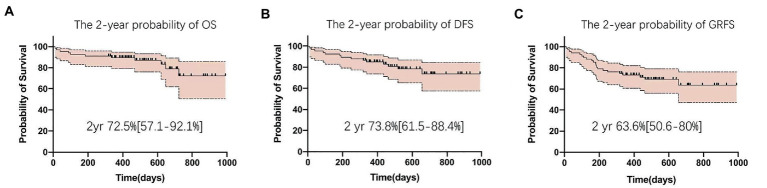
Clinical outcomes after Haplo-PBSCT. **(A)** The 2-year probability of disease-free survival (DFS). **(B)** The 2-year probability of overall survival (OS). **(C)** The 2-year probability of GVHD and relapse-free survival (GRFS).

The 2-year probabilities of DFS and OS were 73.8% (95%CI, 61.5 ~ 88.4%) and 72.5% (95% CI, 57.1 ~ 92.1%), respectively (Figures 6A,B). Overall, 47 patients remained alive and free of relapse, graft failure, III-IV aGVHD, and mod/sev cGVHD with 2-year GRFS of 63.6% (95%CI, 50.6 ~ 80%, Figure 6C and Supplementary Table S1).

## Discussion

GVHD is a major complication of allo-HSCT, particularly when MUD and Haplo donors are involved. CNIs remain the mainstay of GVHD prophylaxis. Previous studies demonstrated the superiority of combining CNI with ATG for prophylaxis of aGVHD and cGVHD in MUD, Haplo, and even MSD settings (15–17).

PT-Cy was also established as an important GVHD prophylaxis strategy. Previous clinical studies demonstrated that PT-Cy combined with other IS, such as CNI, further decreased the incidence of GVHD (10, 18–21). PT-Cy combined with two IS significantly reduced extensive cGVHD and improved the OS and GRFS, on the condition that patients underwent allo-HSCT from MSD or MUD donors (10). Moreover, addition of *in vivo* T cell depletion agent, ATG, to PT-Cy was independently associated with reduced risk of cGVHD and extensive cGVHD. Both strategies did not increase the relapse rate (22, 23). In the retrospective analysis, ATG (2.5 mg/kg on day −1 or day −2 to day −1) combined with PT-Cy decreased incidence of aGVHD to 12% (*p* = 0.029) and incidence of NRM to 8% (*p* = 0.005) with an improved OS (*p* = 0.029) (23). Various strategies were devised to combine ATG and PT-Cy in GVHD prophylaxis as summarized in Table 4 (24–26). Law et al. (24) reported a protocol of ATG 4.5 mg/kg from day −3 to day −1, followed by PT-Cy 50 mg/kg from day +3 to day +4 and cyclosporine for the prevention of graft rejection and GVHD with PBSCs as grafts. At day 100, the cumulative incidences of aGVHD of any grade and grade III-IV were 38.3 and 5.2%, respectively. No case of mod/sev cGVHD was recorded, and the incidence of mild cGVHD was 15.5%. Wang et al. (25) reported a protocol of high dose ATG (10 mg/kg from day −5 to day −2) and cyclosporine, followed by low-dose PT-Cy (14.5 mg/kg from day +3 to day +4) for the prevention GVHD in patients with haplo donors. Contrarily, other studies reported the outcome of low dose ATG (4.5 ~ 5 mg/kg) combined with single dose of PT-Cy (50 mg/kg on day +3) or standard PT-Cy combined with MMF/CsA in haplo-setting (Table 4) (26, 27). Both studies reported that the incidences of grade III-IV aGVHD (6.9 ~ 16) and mod/sev cGVHD (16%) were low.

**Table 4:** GVHD prophylaxis protocols: ATG combined with PT-Cy.

Group	Patients’ features	Protocols	Graft & Donor	aGVHD		All cGVHD	Mod-Sev cGvHD	CIR	NRM	Viremia		OS	DFS	GRFS
				II-IV	III-IV					CMV	EBV			
RJH *N* = 67	Med age 45; AML/MDS/ALL	PTCy (100) + FK506 ± ATG (2.5)	PBSCs Haplo	7.5%	0%	25.4% (1y)	11.9% (1y)	4.5% (1y)	9.0% (1y)	50.7% (100d)	6.0% (100d)	89.5% (1y)	85.0% (1y)	73.1% (1y)
PMCC(24) *N* = 50	Med age 37; AML/ALL/ MDS/CML	ATG (4.5) + CsA + PTCy (100)	PBSC Haplo	38.3%	5.2%	15.5% (6 m)	/	16.0% (1y)	38.2% (1y)	74.0%	61.8%	48.1% (1y)	/	/
PKU(25) *N* = 114	Med age 27; AML/ALL/ MDS/CML	ATG (10) + CsA/MTX/MMF + PTCy (29)	BM + PBSC Haplo (mother/collateral relatives)	26%	5%	30% (2y)	17% (2y)	13% (2y)	6% (2y)	74% (100d)	21% (100d)	83% (2y)	81% (2y)	63% (2y)
SGH(26) *N* = 32	Med age 37; MDS/CMML/ AML/ALL	ATG (5) + PTCy (50)	PBSC + UCB Haplo	19.4%	6.9%	18.8% (1y)	18.8% (6 m)	25.1% (1y)	9.4%	37.5% (180d)	40.6% (180d)	78.4% (1y)	59.0% (1y)	/
SAH(27) *N* = 80	Med age 52 hematological malignancies	ATG (5.0) + PTCy (100) + CsA + MMF	PBSC Haplo	30%	16%	32% (2y)	16% (2y)	26% (1y)	26% (1y)	65% (1y)	50% (1y)	53% (2y)	47% (2y)	/
DNSH(28) CTLA4Ig-DLI group, *N* = 75; DLI group, *N* = 50	Med age 19 hematological malignancies	PTCy (100) + CSA + CTLA4Ig-DLI	PBSC Haplo	9.6%	/	15.3% (1y)	/	/	4%	/	/	/	/	76.6%
	Med age 26 hematological malignancies	PTCy (100) + MMF + CSA + DLI	PBSC Haplo	18.8%	/	36.5% (1y)	/	/	14.4%	/	/	/	/	46%

RJH, Rui Jin Hospital; SGH, Shanghai General Hospital; PKU, Peking University; PMCC, Princess Margaret Cancer Centre; SAH, Saint Antoine Hospital; DNSH, Dharamshila Narayana Superspeciality Hospital; ACBDC, Aflac Cancer and Blood Disorders Center.

In our center, we used a different approach to combine PT-Cy with post-engraft low-dose ATG. The rationale was based on our previous phase II study using PTCy-CsA as GVHD prophylaxis in patients undergoing allo-HSCT with PBSC and myeloablative conditioning. The incidence of aGVHD remained high in MUD or Haplo donor. To tackle the issue, we replaced the CsA with tacrolimus and add a single low-dose of ATG (2.5 mg/kg) after neutrophil engraftment in haplo settings. The rationale for post-engraftment ATG was to avoid the potential impact of immunosuppression drugs used before PTCy. In our previous report, the overall incidence of grade II-IV aGVHD was 9.1 ± 6.1% with 1-year mod/sev cGVHD at 4.6 ± 4.4% in patients undergoing PBSC transplantation in lymphoid malignancies (12). In this study, we extended the analysis to three prospective clinical trials using the same GVHD prophylaxis protocol in patients with myeloid and lymphoid malignancies receiving haplo-donor transplantation only. We observed a low incidence of grade II-IV aGVHD (7.5 ± 3.2%) and no case of III-IV aGVHD at day 100. Besides, all patients with grade II aGVHD responded well to first-line treatment, and no case of steroid-resistant aGVHD was documented. Additionally, the 1-year incidences of cGVHD and mod/sev cGVHD were acceptable. This was the first attempt to add low-dose ATG after engraftment to the GVHD prophylaxis and the short-term outcome was satisfactory with low incidence of aGVHD and particularly no patient died of aGVHD.

More recently, very low incidence of aGVHD was reported using new strategy such as T cell co-stimulation blockade by CTLA4Ig in both MUD or haplo donor transplantation (28, 29). In haplo setting, for patients receiving prophylactic donor lymphocyte (pDLI) infusion with CTLA4Ig based GVHD prophylaxis (*n* = 75), the incidences of aGVHD was low at 9.6% while the NRM and disease progression were low as 4 and 15.7%, respectively. Overall, the 2-year GRFS was significantly improved to 76.6%. Based on these promising data, randomize studies are warranted to confirm the superiority of T cell co-stimulation blockade or PTCy combined with post-engraftment low-dose ATG to conventional PTCy strategy.

One major concern of this enhanced GVHD prophylaxis was the potential infection associated with immunosuppression. We observed a relative high incidence of CMV reactivation (~50%), which was slightly lower than the GVHD prophylaxis with high-dose ATG (10 mg/kg) or at least comparable to that of protocols with moderate dose of ATG (4.5 ~ 5.0 mg/kg) combined with PT-Cy (Table 4). Regarding EBV reactivation and associated PTLD, we observed a low incidence of EBV reactivation, and all these cases were clinically non-significant. The overall incidence of other events of infection was not significantly increased, compared to that of our historical control.

This study had few limitations, which included the small sample and relatively short follow-up period. cGVHD and long-term outcomes, particularly risk of late relapse should be further evaluated. Besides, several other questions, such as the optimal dose and timing of ATG, which were beyond the reach of this study, warrant future clinical trials.

In conclusion, this study demonstrated that standard PT-Cy combined with tacrolimus and additional low-dose post-engraftment ATG in myeloablative Haplo allo-HSCT with PBSCs as grafts was feasible with satisfactory short-term outcomes, in terms of low incidence of aGVHD and NRM.

## Data availability statement

The original contributions presented in the study are included in the article/Supplementary material, further inquiries can be directed to the corresponding authors.

## Ethics statement

The studies involving human participants were reviewed and approved by the Human Ethics Committee of Rui Jin Hospital. The patients/participants provided their written informed consent to participate in this study. Written informed consent was obtained from the individual(s) for the publication of any potentially identifiable images or data included in this article.

## Author contributions

JH, DB, and J-lJ designed the clinical trial and revised the manuscript. W-hG, J-yZ, and L-nW collected the clinical data and wrote the manuscript. LW also helped to collect the clinical data. MW and RD did the statistical analysis. All authors contributed to the article and approved the submitted version.

## Conflict of interest

The authors declare that the research was conducted in the absence of any commercial or financial relationships that could be construed as a potential conflict of interest.

## Publisher’s note

All claims expressed in this article are solely those of the authors and do not necessarily represent those of their affiliated organizations, or those of the publisher, the editors and the reviewers. Any product that may be evaluated in this article, or claim that may be made by its manufacturer, is not guaranteed or endorsed by the publisher.
