# Supplementary material for: Post-transplantation cyclophosphamide combined with tacrolimus and low-dose post-engraftment anti-thymoglobulin as GVHD prophylaxis for patients undergoing peripheral blood stem cell transplantation from haploidentical family donor: A single center analysis
Source: Front Med (Lausanne). 2023 Mar 31;10:1140217. doi: 10.3389/fmed.2023.1140217 (PMC10103611; doi:10.3389/fmed.2023.1140217)
Supplement: Supplementary file 1 [file Data_Sheet_1.docx]

**Table S1: Summary of the transplantation outcomes**

|  | D100 | D180 | 1 year | 2 year |
| --- | --- | --- | --- | --- |
| All aGVHD | 14.9±4.4% | 14.9±4.4% | / | / |
| II-IV aGVHD | 7.5±3.2% | 7.5±3.2% | / | / |
| cGVHD | / | / | 25.4±5.4% | 25.4±5.4% |
| Mod/sev cGVHD | / | / | 11.9±4.0% | 11.9±4.0% |
| NRM | 7.5±3.2% | **/** | 9.0±3.5% | 9.0±3.5% |
| CIR | **/** | **/** | 4.5±2.5% | 16.0±6.4% |
| DFS | **/** | **/** | 85% (73.4~89.8%) | 73.8% (61.5~88.4%) |
| OS | **/** | **/** | 89.5% (76.0~92.1%) | 72.5% (57.1~92.1%) |
| GRFS | / | / | 73.1%(63.0-82.5%) | 63.6%(50.6-80.0%) |
